# NMRium: Teaching nuclear magnetic resonance spectra interpretation in an online platform

**DOI:** 10.3762/bjoc.20.4

**Published:** 2024-01-05

**Authors:** Luc Patiny, Hamed Musallam, Alejandro Bolaños, Michaël Zasso, Julien Wist, Metin Karayilan, Eva Ziegler, Johannes C Liermann, Nils E Schlörer

**Affiliations:** 1 Zakodium Sàrl, Route d'Echandens 6b, 1027 Lonay, Switzerland; 2 Faculty of Chemistry and Earth Sciences, Friedrich Schiller University, 07743 Jena, Germany; 3 Chemistry Department, Universidad del Valle, Cali 76001, Colombia; 4 Australian National Phenome Center, and Center for Computational and Systems Medicine, Health Futures Institute, Murdoch University, Harry Perkins Building, Perth WA6150, Australia; 5 Department of Chemistry, Case Western Reserve University (CWRU), Cleveland, Ohio 44106, United States; 6 Department of Chemistry, Johannes Gutenberg University Mainz, 55099 Mainz, Germany

**Keywords:** chemical education, E-learning, NMR, NMR spectra, structure elucidation

## Abstract

NMRium is the first web-based software that allows displaying, processing, interpretation, and teaching of 1D and 2D NMR data in a user-friendly interface. It can import the most common data formats (e.g., JCAMP-DX, Bruker, Varian, and Jeol). While the scope for the use of NMRium encompasses a variety of applications such as being a component in data repositories or electronic lab notebooks (ELN), performing structure elucidation or preparing raw spectral data for publication, it also excels in enhancing teaching of NMR interpretation. In this paper, we present some current possibilities of this new tool. Several series of exercises are already provided on https://www.nmrium.org/teaching.

## Introduction

For the validation of molecular structures, nuclear magnetic resonance (NMR) spectroscopy is an indispensable methodology in the daily routine of synthetic chemistry laboratories. Arguably, NMR experiments serve as the ‘eye of the synthetic chemist’ because they allow a rapid and comparatively easy deduction of structural features with the only requirement being the molecule's solubility in deuterated solvents. Therefore, NMR spectroscopy is of ubiquitous presence in many disciplines today. At the same time, with the high throughput analysis of samples that has been a common approach in academic laboratories, the ability of chemists to conduct the interpretation of experimental results becomes crucial. In addition, NMR spectroscopy is one of the methods that has been delivering digital experimental raw data for a long time. However, the way the spectra are interpreted and published in the area of organic chemistry has been almost unchanged over the last five decades, in spite of tremendous experimental progress [1]. Only in specialized (sub)disciplines of NMR (e.g., biomolecular and metabolomic applications), adequate handling of spectral data has been adapted or is currently under development [2–5].

A fundamental portion of the aforementioned misadjustment roots in outdated manners of teaching NMR: Besides the fact that 2D experiments play only a minor role in published routine analyses of organic molecules [6–11], the way to provide data in classes and exercises is usually based on a ‘static’ (i.e., printed/pdf-style) presentation of NMR spectra. No interactive data handling is possible, and manual annotations will typically be made during practicing exercises. Advanced approaches require the availability of locally installed (oftentimes commercial) software, which imposes additional preconditions that cannot easily be overcome in many institutions [12–13].

**Figure 1:**
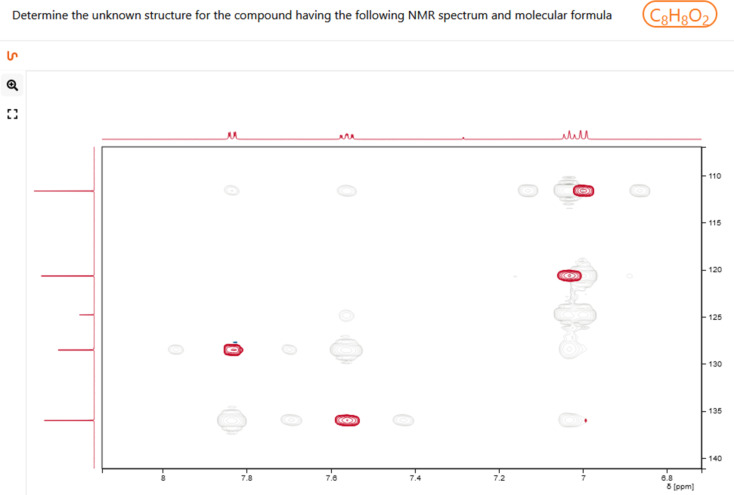
NMRium.org user interface showing a 2D NMR quiz for an unknown compound with a given molecular formula.

Many tasks that required locally installed software before can now be easily performed using web applications (e.g., cloud office services), making the web browser a powerful platform for specialized data handling applications. This trend has not been withheld from science: With the advent of web tools for the online representation and manipulation of scientific data [14–17] and the development of software for spectroscopy visualization [18–26], new possibilities to use web-based strategies for teaching evolved [27–29]. In this context, we provide undergraduate students with a series of interactive exercises of varying difficulty that are solved directly from a web browser (Figure 1). Students now have access to a tool allowing them the same techniques used in research to solve problems of increasing difficulty. In this paper, we describe the online tool NMRium (can be accessed through NMRium.org), exploring for educational purposes when teaching NMR spectroscopy in chemistry.

## Results and Discussion

At the core of NMRium is an open source web component [30–31]. It is derived from a freely available chemoinformatics tool set directed at scientific and educational applications [32–33]. It is a tool to display spectral data and metadata that has been developed as part of our efforts to aid better handling and evaluation of NMR data. While initially intended for small molecules data repositories and databases [34–35], NMRium provides unique functionalities that make it particularly well-suited for educational application.

### Teaching the interpretation of NMR spectra

Nowadays, the interpretation of NMR experiments is part of the curriculum for chemistry students. However, NMR spectra usually remain restricted to a non-interactive presentation, most of the time in the form of plots or pdf depictions, without the student being involved actively in the data manipulation. In contrast, NMRium not only allows the dynamic visualization of NMR experiments in presentations, but also facilitates the integration of 2D experiments in teaching. This aspect is oftentimes disregarded, although those experiments contribute a superior part of the information in spectra interpretation and are standard for today’s instrumentation [32–33]. Thus, the first part of data handling, i.e., processing and preparation of an interpretation-ready state of spectra, is insufficiently treated in education. In the same way, the export format of evaluated/assigned experiments remains vague [36], and adequate recent developments for electronic publication formats [37] are hardly considered.

Therefore, the following aspects and their consideration when teaching assisted by NMRium will be discussed:

- Preprocessing and Fourier transformation
- Interactive spectra manipulation
- Structural assignment and electronic bookkeeping
- Many import and export options and storing entire NMRium projects in their current state, whether partially or fully analyzed.

### Features of NMRium

What differentiates NMRium from most other NMR data processing software, specifically with respect to its educational application, is the ability to handle NMR data (proprietary format of various manufacturers, JCAMP-DX standard files, or NMReDATA format) independently of the operating system by just dragging and dropping the data in a browser-based web tool. Despite being a JavaScript-based web tool, an online connection is not required after the software was loaded as the handling of spectra happens locally in the browser. Projects can be stored at any point of processing/assignment as a downloadable '.nmrium' file, thus allowing a later continuation of data handling or importing of a fully processed and assigned data set, by a simple drag-and-drop procedure. Besides various processing options (so far only available for 1D spectra), the interface also includes an editor for molecule structures which can be directly used to assign NMR signals. A documentation for all steps of a standard processing and assignment procedure, including import/export options, can be found on the project website (as well as in the supplementary information) and is continuously extended. In addition, NMRium features online assistance with short movies.

For teaching, the web-based nature makes the software an outstanding tool for live instruction: Steps demonstrated by the instructor may be synchronously reproduced by participants by simply sharing a URL. Also, the teaching scientist may work/demonstrate using a ‘solution’ project for demonstration, while students elaborate on a ‘problem-solving’ project which contains a different level of information. There are three predefined modes (‘workspaces‘) that bundle functionalities from the accordion panels for different purposes and custom modes can also be defined.

**Figure 2:**
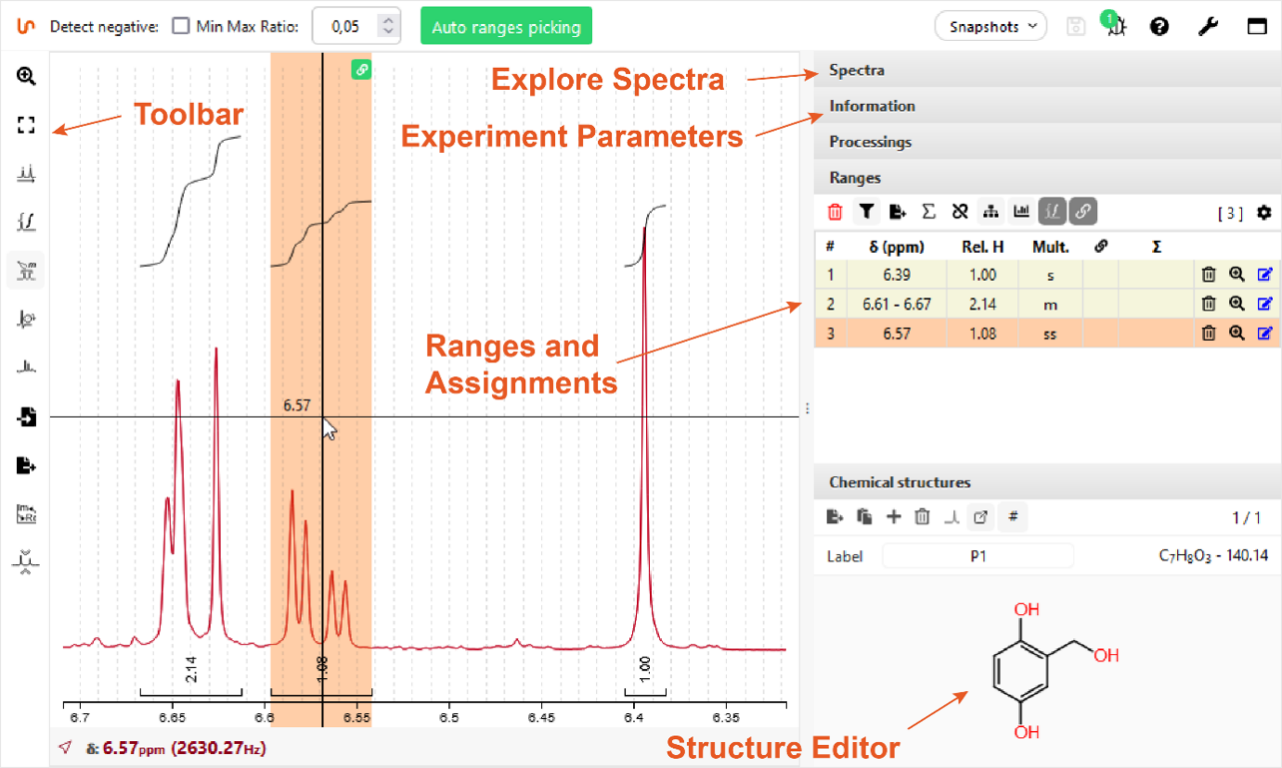
NMRium interface assignment functionality for a drag-and-drop example.

In the same way, the web examples for teaching provided on the project homepage [38] offer a range of options for the development of structure-solving skills (Figure 2), and one’s own examples including 1D and 2D experiments, with and without assignment, can be easily prepared for in class presentations or video conferencing.

### Scenarios and examples for teaching with NMRium

NMRium is a versatile tool for teaching NMR spectroscopy, including spectrum management, processing, and analysis, applicable across various areas and proficiency levels. The subsequent section will provide practical illustrations, with selected examples that demonstrate its utility.

#### Teaching the processing of 1D NMR spectra

Fourier transformation (FT) of NMR data and standard operations like apodization/window functions and zero-filling are now applied automatically by common software packages using presets. While this provides convenience, it also leads to a loss of knowledge for new users on their impact on properties such as resolution and signal-to-noise ratio and the balance between them.

For teaching labs (practical laboratory classes including examples of synthesis and compound characterization), NMRium provides a full 1D FT workflow and therefore an ideal possibility to introduce basic concepts of data processing, data presentation, and data evaluation. Spectra from most NMR instruments may be directly used (Bruker, Jeol) without conversion and then can be subjected to an educational protocol of FT, phase correction (manual or automatic), baseline correction, and signal analysis by peak picking and integration. Students can interactively observe the effect of the parameters on their spectra as all operations are recorded as “filters” whose arguments may be reversed or changed afterwards to alter the appearance of the processed spectrum [39].

**Figure 3:**
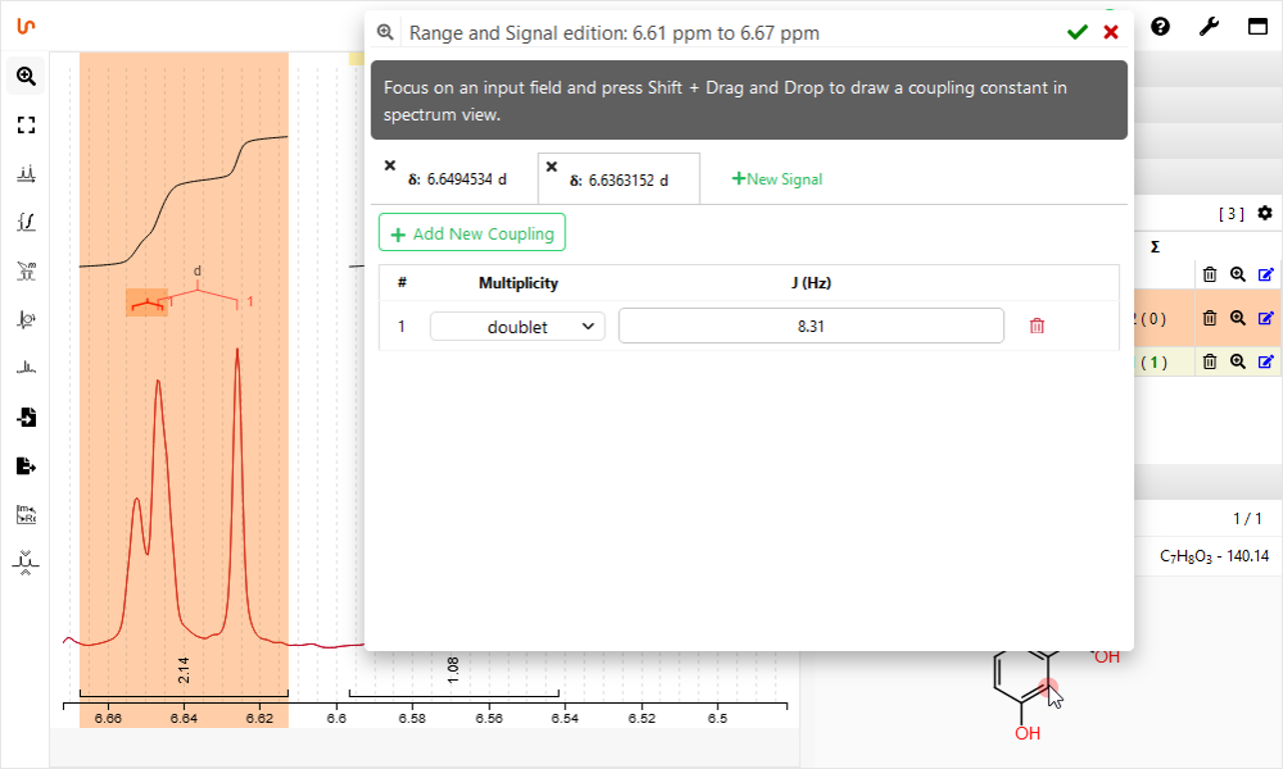
Analysis of overlapping signals with the NMRium ranges editor.

Alternative to performing peak picking and integration separately, NMRium provides the ‘ranges’ picking (Figure 2), a combined tool for signal selection, integration, and automatic multiplet analysis even for overlapping resonances (Figure 3), which then can be used for class assignments to assign signals and atoms in the integrated structure editor. All these manipulations of the processed spectra can be demonstrated in classes while at the same time hands-on practiced by the students. During assignment, NMRium is able to recognize topicity and, e.g., distinguish diastereotopic protons.

NMRium also offers modes for multiple spectra display: It allows to overlay or stack a series of 1D spectra for comparison (e.g., to demonstrate the spectral differences between a reaction substrate and product or analysis of kinetic studies that include several data points) or overlaying of 2D spectra, for example, to show the different outcomes of related experiments such as COSY/TOCSY or HSQC/HMBC. Students will be enabled to learn these online comparison aspects by overlaying 1D or 2D data easier than by a simple comparison of spectra individually.

Finally, the whole workspace including 1D and 2D spectra, annotations, and assignments can be saved in JSON format, thus allowing to conserve and retrieve work in progress at any point. Several other output formats for spectra and signals are available, including a string format typical for publications, giving students an incentive to use the tool for their spectra reports in the lab. An illustrated reference manual with step-by-step explanations of typical NMR processing workflows is also available [40].

#### Using available spectra for quiz sessions/exercises for teaching classes on structure elucidation

Another valuable feature is predefined online exercises, which are accessible through the homepage. When learning how to analyze NMR spectra, one important feature which cannot be provided by classical, ‘static’ exercises (printed on paper or distributed as pdf graphs) is the possibility to interactively examine the signals (Figure 2 and Figure 3). NMRium offers these functions in a selection of example quizzes for structure elucidation with varying levels of difficulty, all of them including a molecular formula: Starting from a set of (currently 23) one-dimensional ^1^H NMR experiments [41], ranging from simple to advanced, spectra may be interactively transformed (enlargement, zoom, distance measurement in Hz) and signals can be integrated, to allow students to identify the compound sought-after. For more advanced students, examples with combined 1D, 2D, and even some heteronuclear spectra can be used [42]. Here, one set including eight examples consisting exclusively of one-dimensional ^1^H and ^13^C NMR spectra and eight additional exercises including a combination of ^1^H, ^13^C, COSY, HSQC, and HMBC experiments provide quiz material for intermediate to more advanced students. A third group of exercises includes additional experiments e.g., NOESY or 1D NOE spectra and 1D/2D heteronuclear experiments including nuclei such as ^15^N or ^19^F [43]. For all three exercise collections, students may draw a derived structure and control online if their suggestion is correct.

#### Building one’s own exercises

The options presented before do not involve the entire potential of NMRium for teaching. Lecturers may want to include peak picking, or for example, for assignment purposes, ranges/zones picking and the possibility to link signals and atoms in a structure. Those options can be individually restricted or added by disclosing or locking panels of the accordion menu when designing exercises. More importantly, however, there is also complete freedom of the number and combination of individual NMR spectra that are included in an exercise. Any combination of 1D, 2D, homo- and heteronuclear experiments is feasible to create new challenges.

Creating new exercises is very simple and straightforward [40]. The exercises consist of JCAMP-DX spectra as well as a molfile containing the correct answer. While these files can be hosted anywhere, the easiest way is to use a dedicated GitHub repository. We provide a repository template with a preconfigured action set that will create the JSON and table of content files for the users. The users only have to create a folder structure to define the hierarchy of their exercise set and put the desired spectra in JCAMP-DX format and a molfile of the solution structure in each exercise folder. The rest of the required steps happens automatically after the user saves the changes (“commit” in Git terminology) and you get links to distribute to the students.

NMRium is also designed as a component which can be embedded in other websites for more advanced teaching scenarios.

#### Teaching NMR assignment

Finally, teachers can also give their more advanced students assignments without the online quiz functionality, by providing spectra or a complete .nmrium dataset for the students to process, analyze, and finally assign to a molecular structure. Students can then save the result of their work as .nmrium and submit it to the teacher who can then assess their solution and their solving strategy.

## Conclusion

To the best of our knowledge, the software NMRium presented in this paper provides the first web-based workflow to process, display, evaluate, and assign NMR data for small, organic molecules. It allows local handling of data (only web, but no cloud) and includes training data sets that can be used as exercises: All data loading and processing is done in the user's web browser and do not transit to any server, ensuring the privacy of the data. Additionally, as a free online tool, it offers a wealth of possibilities to interactively teach the interpretation of NMR spectra in chemistry classes and practical courses and can be integrated in other teaching environments. Thus, its easy to grasp functionality would make it a preselected tool for the review process of chemistry publications. Future developments of NMRium include the processing of 2D NMR experiments, as well as a CASE- and prediction-supported assignment tool with an integrated validation function for assigned data [44]. Currently, first implementations of NMRium in other environments have already successfully started.

## Data Availability

The data used for/in this study is openly available in Zenodo at https://doi.org/10.5281/zenodo.10203766.
